# Characterization and genomic analysis of kraft lignin biodegradation by the beta-proteobacterium *Cupriavidus basilensis* B-8

**DOI:** 10.1186/1754-6834-6-1

**Published:** 2013-01-08

**Authors:** Yan Shi, Liyuan Chai, Chongjian Tang, Zhihui Yang, Huan Zhang, Runhua Chen, Yuehui Chen, Yu Zheng

**Affiliations:** 1 School of Metallurgical Science and Engineering, Central South University, Changsha, 410017, PR China; 2 Chinese National Engineering Research Center for Control & Treatment of Heavy Metal Pollution, Changsha, 410017, PR China

**Keywords:** *Cupriavidus basilensis* B-8, Kraft lignin, Biodegradation, Characterization, Genomic analysis

## Abstract

**Background:**

Lignin materials are abundant and among the most important potential sources for biofuel production. Development of an efficient lignin degradation process has considerable potential for the production of a variety of chemicals, including bioethanol. However, lignin degradation using current methods is inefficient. Given their immense environmental adaptability and biochemical versatility, bacterial could be used as a valuable tool for the rapid degradation of lignin. Kraft lignin (KL) is a polymer by-product of the pulp and paper industry resulting from alkaline sulfide treatment of lignocellulose, and it has been widely used for lignin-related studies.

**Results:**

Beta-proteobacterium *Cupriavidus basilensis* B-8 isolated from erosive bamboo slips displayed substantial KL degradation capability. With initial concentrations of 0.5–6 g L^-1^, at least 31.3% KL could be degraded in 7 days. The maximum degradation rate was 44.4% at the initial concentration of 2 g L^-1^. The optimum pH and temperature for KL degradation were 7.0 and 30°C, respectively. Manganese peroxidase (MnP) and laccase (Lac) demonstrated their greatest level of activity, 1685.3 U L^-1^ and 815.6 U L^-1^, at the third and fourth days, respectively. Many small molecule intermediates were formed during the process of KL degradation, as determined using GC-MS analysis. In order to perform metabolic reconstruction of lignin degradation in this bacterium, a draft genome sequence for *C. basilensis* B-8 was generated. Genomic analysis focused on the catabolic potential of this bacterium against several lignin-derived compounds. These analyses together with sequence comparisons predicted the existence of three major metabolic pathways: *β*-ketoadipate, phenol degradation, and gentisate pathways.

**Conclusion:**

These results confirmed the capability of *C. basilensis* B-8 to promote KL degradation. Whole genomic sequencing and systematic analysis of the *C. basilensis* B-8 genome identified degradation steps and intermediates from this bacterial-mediated KL degradation method. Our findings provide a theoretical basis for research into the mechanisms of lignin degradation as well as a practical basis for biofuel production using lignin materials.

## Background

The world has been confronting an energy crisis due to depletion of finite fossil fuel resources [1]. Furthermore, an ever-increasing level of greenhouse pollution from the combustion of fossil fuels in turn aggravates global warming and climate change [2]. This has led to a realization that modern society must turn to renewable forms of energy and chemical production. Hence, there is considerable interest in the utilization of plant biomass for the production of bioenergy and renewable chemicals [3].

Lignin is the most abundant aromatic compound on earth and is second only to cellulose in its contribution to living terrestrial biomass [4]. It is a complex aromatic heteropolymer comprised of phenylpropanoid aryl-C_3_ units linked via a variety of ether and carbon-carbon linkages, and it is recalcitrant to microbial degradation [5]. The biological degradation of lignin is not only one of the most important parts of the biospheric carbon and oxygen cycle [6], but it is also a central aspect for industrial use of cellulosic biomass, such as bioethanol production and manufacture of cellulose-base chemicals and materials [7].

Despite lignin’s natural recalcitrance, some of fungi are able to decompose lignin. The best-characterized degraders are white-rot fungi, in particular *Phanerochaete chrysosporium*[8]. In nature, phenol oxidases including lignin peroxidase (LiP), manganese peroxidase (MnP), and laccase (Lac) are secreted by white-rot fungi and are assumed initially to attack lignin. These enzymes act through radical reactions [9]. Although fungi are the main contributors to lignin degradation, bacteria display versatile pathways to degrade aromatic substances, from simple phenols to highly complex lignins and related xenobiotic substances. Furthermore, some low molecular weight compounds (mostly aromatic carboxylic acids) formed from fungal lignin degradation may be further metabolized by bacteria [10]. Several bacteria strains such as *Streptomyces viridosporus* T7A [11], *Nocardia*, and *Rhodococcus*[12] have been reported to degrade lignin. The actual catabolic pathways of lignin derivatives and the responsible enzymes and genes have been investigated using molecular methods in a few bacterial strains, including *Sphingomonas paucimobilis* SYK-6 [9]. However, bacterial genomic analysis of lignin degradation pathways is still lacking. Comprehensive elucidation of the bacterial genes and enzyme systems for lignin degradation is important for understanding the process of the earth’s carbon cycle and for providing useful tools for the conversion of lignin into intermediate metabolites of industrial value [9]. The structure of natural lignin is very complex, and intact lignin is not commercially available. However, because of the similarities to natural lignin, kraft lignin (KL) has been widely used for lignin-related studies [13-17].

In our previous research, the novel beta-proteobacterium strain *C. basilensis* B-8 was isolated from steeping fluid of the erosive bamboo slips derived from Kingdom Wu during the Three-Kingdoms Dynasty of ancient China (A.D. 220–280). This bacteria was found to degrade KL and related aromatic compounds. However, its gene characteristics and the mechanisms by which it degrades KL were still unclear. Therefore, the objectives of this current study were to: (i) investigate the capability of this bacterial strain to degrade KL, (ii) identify the genes responsible for lignin degradation, (iii) identify the derivatives of this process, and (iv) reconstruct the metabolic pathways involved in degradation of lignin and generation of its derivatives.

## Result and discussion

### Optimization of temperature and pH on KL degradation by *C. basilensis* B-8

**Figure 1:**
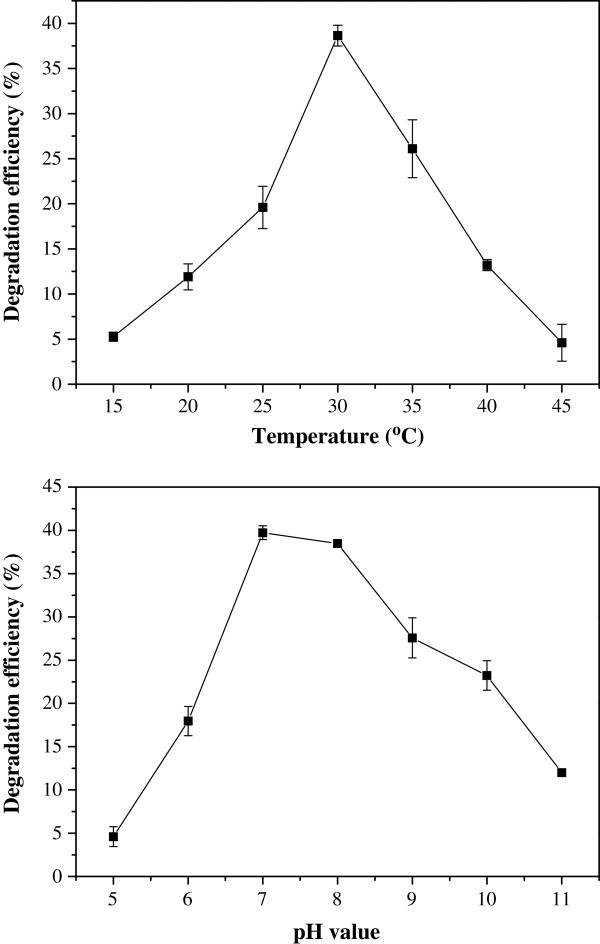
**Effect of temperature and pH on KL degradation by *C. basilensis *B-8. (a)** temperature and** (b)** pH. Average values of three replicates are shown with the standard error of the mean as error bars.

According to the actual results, the temp optimum was 30°C and the pH optimum was between 7–8.5 (Figure 1), as presented in previous reports, e.g. the optimum pH of *Aneurinibacillus aneurinilyticus* is 7.6 [17] and the corresponding values for *Comamonas* sp. B-9 and *Bacillus* strain are 7 [18] and 7.6 [19], respectively. For *Streptomyces* strains, the optimal pH ranges from 7.8–8.5 [20].

### Bacterial growth and KL degradation

**Figure 2:**
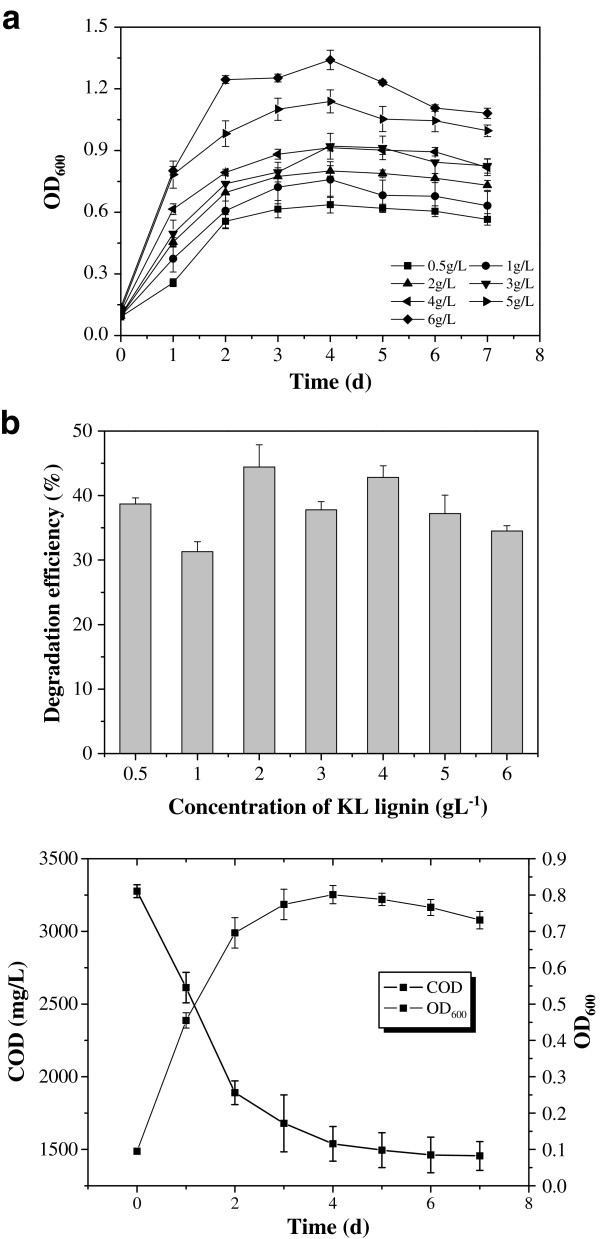
**Bacterial growth and KL degradation in different initial concentration of KL. (a)** Bacterial growth in different initial concentration, **(b)** KL degradation rate at seventh day in different initial concentration, **(c)** Bacterial growth and COD reduction in 2 g · L^-1^ KL. Average values of three replicates are shown with the standard error of the mean as error bars.

The rate of *C. basilensis* B-8 growth was evaluated under seven different initial KL concentrations ranging from 0.5 g L^-1^ to 6 g L^-1^. *C. basilensis* B-8 grew well under initial concentrations from 1 g L^-1^ to 6 g L^-1^ (Figure 2a), indicating that bacterial growth would not be inhibited under the tested concentrations. However, the optical density (OD) value of the cultured sample increased with the increase in initial KL concentration. The rates of KL degradation under different initial concentrations all surpassed 31% on day 7 (Figure 2b), but there was no obvious correlation between the initial concentration and the KL degradation rate. The highest KL degradation rate of 44.4% was observed at an initial concentration of 2 g L^-1^, and the largest KL degradation capacity of 2.1 g L^-1^ was observed at the initial concentration of 6 g L^-1^.

The growth and KL degradation capacity of *C. basilensis* B-8 in nutrient medium with a KL concentration of 2 g L^-1^ were investigated in detail. The results are shown in Figure 2c. *C. basilensis* B-8 growth was substantially faster during the first 2 days and reached the maximum at day 4. KL degradation mainly occurred during the initial 2 days. Accordingly, the maximum KL degradation rate of 722.8 mg L^-1^ Day^-1^ was recorded during this period. From the third day, KL degradation was continuous, but the degradation rate was decreased. The chemical oxygen demand (COD) value reached 1455.4 mg L^-1^ from the initial 3276.4 mg L^-1^ at day 7. The growth and KL degradation observed in this experiment were different from those of *Citrobacter* strains, which must initially use glucose and peptone as carbon sources and subsequently utilize lignin as a co-metabolite [21]. Accordingly, KL could be the sole nutrition source of *C. basilensis* B-8, as it must be metabolized during the initial growth stage to provide carbon and energy for growth. A similar process of KL degradation was also reported for *Comamonas* sp. B-9 [18] and *Streptomyces viridosporus*[11], which also showed a great capacity for KL degradation. This predicts that bacteria that use lignin as their sole carbon source must metabolize it throughout the whole life cycle; therefore, the efficiency and total amount of lignin degradation would be relatively higher for these strains than those using lignin as co-metabolite.

### Analysis of enzymes and genes related to KL degradation

**Figure 3:**
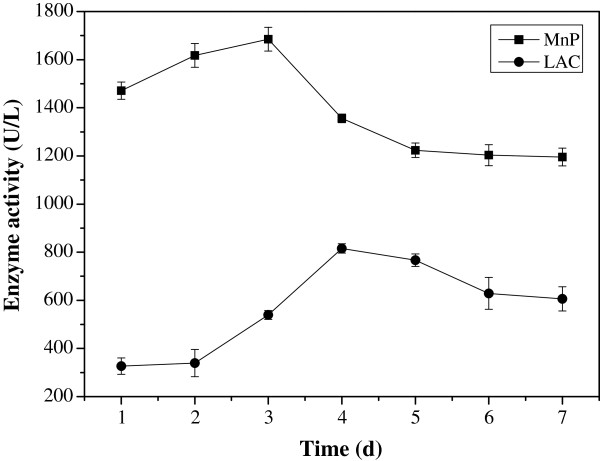
**The Activity of MnP and Lac during 7 days incubation.** Average values of three replicates are shown with the standard error of the mean as error bars.

Three major enzymes including LiP, MnP, and Lac, which use low-molecular-weight mediators to carry out lignin degradation, have been well characterized in microorganism [22]. The activity of these three enzymes from *C. basilensis* B-8 is shown in Figure 3. MnP activity increased significantly during the initial 3 days, with a maximum of 1685.3 U/L at day 3, followed by a slight decrease from day 4. Lac activity was maintained at a low level on day 1. A rapid increase was then observed from day 2, with a maximum of 815.6 U/L on day 4. These results indicated that MnP played a crucial role during the entire process of KL degradation by *C. basilensis* B-8, whereas Lac mainly functioned during the latter stages of the reaction. A similar conclusion was also proposed in previous reports [23,24]. However, the mechanism of lignin micro-biodegradation is complicated; thus, a complete explanation requires further study. In addition, no obvious LiP activity was observed during the course of KL degradation, indicating that active LiP was not produced by *C. basilensis* B-8. Similar to this strain, some white-rot fungi and bacteria (i.e., *Dichomitus squalens*, *Lentinula edodes*[10], and *Comamonas* sp. B-9 [18]), which simultaneously produce MnP and Lac, were reported not to secret detectable levels of LiP. These organisms are also strong lignin degraders. Since LiP is responsible for the oxidation of non-phenolic syringyl and biphenyl model compounds (which exist in certain types of lignins, like hardwood) and subsequent ring cleavage [10], it is conceivable that the efficiency of hardwood degradation by *C. basilensis* B-8 and the other microorganisms mentioned above are relatively low.

No LiP activity was detected in this study, and it was not surprising that no gene encoding LiP was identified via genomic analysis of *C. basilensis* B-8 (see supplementary files). However, a genomic search for MnP genes in the genome of *C. basilensis* B-8 rendered only open reading frames (ORFs) with low amino acid (aa) identities of 27.0% and 30.0% with the MnP genes of the fungi *Pleurotus ostreatus* and *Ganoderma australe*, respectively. MnP gene retrieval using the NCBI public database showed that no MnP genes from bacteria were available; furthermore, very few reports detail the presence of MnP enzymes in bacterial systems [25].

### Metabolite characterization via GC-MS

**Figure 4:**
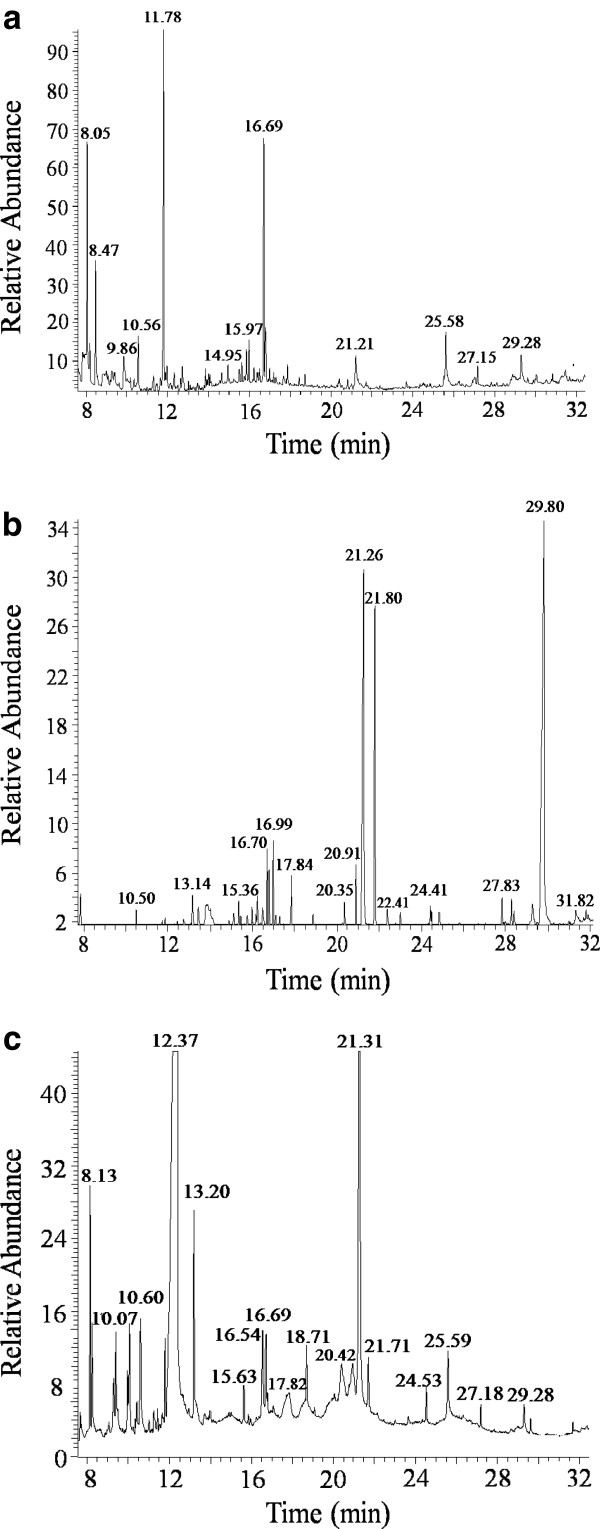
**TIC of TMS derivatives of compounds extracted with trichloromethane from kraft lignin medium incubated with *Cupriavidus *sp.** B-8 **a**: 0d; **b**: 3d; **c**: 6d.

The low molecular weight compounds released from lignin due to KL degradation by *C. basilensis* B-8 were analyzed by GC-MS. The total ion chromatograph (TIC) patterns corresponding to the compounds extracted with ethyl acetate from the control (uninoculated medium sample) and degraded samples are shown in Figure 4a-c, and their peak identity is depicted in Table 1. In the TIC pattern of the control sample (Figure 4a), peaks at RT 8.1 and RT 10.6 were identified as acetic acid and phenol, respectively. The identification of these two important intermediate metabolites generated during the degradation of lignin by microorganisms [3,26] may be attributed to the chemical oxidation of lignin due to aeration and agitation. Moreover, other lignin-related compounds were also indentified, suggesting partial degradation of KL during the industrial production process [27].

**Table 1:** Chromatographic peak identification of metabolic products from KL degradation

***No***	***RT***^***a***^	***Present in***			***Compound***
		**Figure 4a (0d)**	**Figure 4b (3d)**	**Figure 4c (7d)**	
1	8.1	+	-	-	Acetic acid
2	8.5	+	-	-	Methyl acetate
3	10.5	-	+	-	Ethanedioic acid
4	10.6	+	-	-	Phenol
5	10.6	-	-	+	2,3-dihydro-3,5-dihydroxy-6-methyl-4-pyrone
6	11.8	+	-	-	3,5-Dimethyl-4-hydroxybenzaldehyde
7	13.1	-	+	-	2-methylnaphthalene
8	15.6	-	-	+	Ethyl gallate
9	16.5	-	-	+	4-hydroxy-3-methoxyacetophenone
10	16.7	+	-	+	Cinnamic acid
11	16.7	-	+	-	2,3-dihydro-5-methylfuran-2-one l
12	16.8	+	+	+	3,5-di-tert-butyl phenol
13	18.7	-	-	+	Gentisate
14	21.3	-	-	+	4-hydroxy-3,5-dimethoxy acetophenone
15	21.7	-	-	+	3,4-dihydroxyphenylacetic acid
16	21.8	-	+	-	4-Hydroxycinnamic acid
17	22.4	-	+	-	Methyl 3-(α,α,-dimethyl)propionate
18	24.4	-	+	-	Octadecanal
19	25.6	+	-	+	hexadecanoic acid
20	27.2	-	-	+	3,5-dihydroxybenzoic acid
21	29.8	-	+	-	hexadecamide

The number of peaks in the TIC increased significantly after 3 and 6 days of incubation with *C. basilensis* B-8 as compared to the control. Many low molecular weight compounds such as 2, 3-dihydro-3, 5-dihydroxy-6-methyl-4-pyrone; 3, 5-dimethylbenzaldehyde; 2-methylnaphthalene; cinnamic acid; and gentisate were identified in the extract of the degraded sample (Figure 4b-c and Table 1), and these were not present in the extract of the control sample. The detected guaiacol-related compound and cinnamic acid could be easily related to the oxidation of the guaiacyl units from precursor coniferyl alcohol and ρ-hydroxyphenyl units generated from precursor ρ-coumary alcohol. These are considered to be basic moieties with syringyl units from precursor sinapyl alcohol that are components of the lignin structure [3]. Unfortunately, no syringyl-related compounds were identified in the sample. In addition to aromatic compounds, more acid-type compounds were identified than aldehyde and ketone-type compounds due to degradation of lignin. A similar study was also performed previously in *Aneurinibacillus aneurinilyticus*[17]. The low molecular weight compounds identified in the extracts of the inoculated sample favor the conclusion that KL was degraded by *C. basilensis* B-8.

### The initial degradation of lignin into low molecular weight compounds by extracellular phenoloxidases

The extracellular oxidative enzymes LiP, MnP, and Lac are defined as phenoloxidases, which are responsible for the initial degradation of lignin and have been intensively studied in fungi. Although enzymology of bacterial lignin degradation has not been as thoroughly investigated as that of fungi, there are indications that bacteria use extracellular peroxidase for lignin degradation [11]. The activities of MnP and Lac from *C. basilensis* B-8 have been observed using colorimetric enzyme assays (Section “Analysis of enzymes related to KL degradation”). These observations indicted the existence of a novel MnP or its isozyme in *C. basilensis* B-8. The further study, purification, and characterization of these enzymes is currently under way.

High MnP activity has also been documented in a previous report investigating *Citrobacter* strains [21] that need glucose as an extra carbon source to produce hydrogen peroxide, which serves as a cosubstrate for the ligninolytic activity of MnPs via glucose oxidation. However, glucose was not involved in the lignin degradation by *C. basilensis* B-8; in addition, the genes encoding glyoxal oxidase and aryl alcohol oxidase that are responsible for hydrogen peroxide production in fungi were not found in *C. basilensis* B-8, suggesting hydrogen peroxide in *C. basilensis* B-8 is generated via other unknown mechanisms. The genomic search of *C. basilensis* B-8 for a Lac gene only indicated one ORF with low aa identity (32.0%) with the LAC gene from *Thermus thermophilus* HB27. Given that some dye-type peroxidases, which are active against KL and lignin model compounds, have been identified in several bacteria [3], it is reasonable to predict that the detected enzymes form *C. basilensis* B-8 may belong to this group.

### Catabolism pathways for lignin components

Many low molecular weight compounds were produced from initial KL degradation. The enzymes that are involved in catabolic pathways for the degradation of lignin fragments have been identified and characterized in several bacterial [28-30]. Here, three important degradation pathways for lignin basic derivatives, including coumaric acid, ferulic acid, cinnamic acid, phenol, salicylate, and 3-hydroxybenzoate, were predicted basing on genomic analysis. Moreover, all of these compounds were shown to support the growth of *C. basilensis* B-8.

### The *β*-ketoadipate central pathway for coumarate, ferulate, and cinnamate degradation

**Figure 5:**
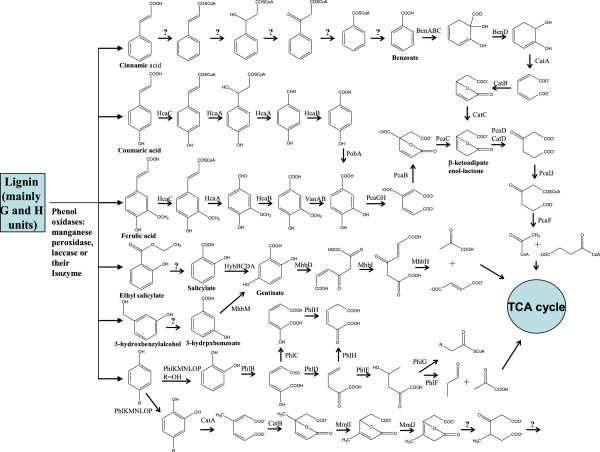
**Catabolic pathways for the catabolism of lignin and its derivatives in *C. basilensis *B-8. ? means that the enzyme encoding such biochemical step is still unknown.**

The central reactions of the *β*-ketoadipate pathway in *C. basilensis* B-8 are shown in Figure 5. The two branches of the *β-*ketoadipate pathway (i.e. the catechol branch encoded by *cat* genes and the protocatechuate branch encoded by *pca* genes) can convert catechol and protocatechuate into the Krebs cycle intermediates succinate and acetyl coenzyme A [31]. Biochemical studies and amino acid sequence data indicated that the enzymes of this pathway are highly conserved among phylogenetically diverse organisms that possess this pathway. 

The *cat* and *pca* gene products of *C. basilensis* B-8 were significantly similar to proteins of known function from other bacteria, mainly *Cupriavidus basilensis* OR16 (Table 2), which has been reported earlier [32]. However, unlike many other bacteria whose *cat* and *ben* genes are usually organized in a single cluster, these genes in *C. basilensis* B-8 were organized in three clusters at different positions (see alignment result in supplementary files). The *catA* gene encoding catechol-1, 2-dioxygenase, which starts the catechol branch of the *β-*ketoadipate pathway, clustered together with the *benABCD* genes that encode the enzymes responcibe for converting benzoate into catechol. This operon was regulated by CatR, a member of the LysR family of regulatory proteins in *P. pudita*[33,34]. There are two copies of the *catC* gene that shared 71.0% aa identity within the genome of *C. basilensis* B-8; one of these genes was located upstream of the *catB* gene and the other was located the downstream of the *catD* gene.

**Table 2:** Genes encoding the β-ketoadipate pathway and peripheral reactions

**Gene (orf no.)**^**1**^	**Gene product**	**Size (aa)**^**2**^	**Function**	**Organism**	**% Identity/aa**	**Accession no.**
*catA* (002058)	CatA	304	Catechol 1,2-dioxygenase	*Lutiella nitroferrum* 2002	80/304	ZP_03698425.1
*catB* (007569)	CatB	142	Muconate cycloisomerase	*Cupriavidus basilensis* OR16	91/382	ZP_09625347.1
*catC1* (007567)	CatC1	92	Muconolactone *δ*-isomerase	*Cupriavidus basilensis* OR16	93/92	ZP_09625348.1
*catC2* (006855)	CatC2	91	Muconolactone *δ*-isomerase	*Alicycliphilus denitrificans* BC	79/92	YP_004127266.1
*catD* (006858)	CatD	273	3-oxoadipate enol-lactonase	*Acidovorax avenae subsp. avenae ATCC* 19860	68/273	YP_004236298.1
*catR* (002057)	CatR	307	Transcriptional activator (LysR family)	*Burkholderia cenocepacia* J2315	83/307	YP_002233374.1
*benA* (002059)	BenA	462	Benzoate dioxygenase large subunit	*Cupriavidus basilensis* OR16	96/462	ZP_09623640.1
*benB* (002060)	BenB	166	Benzoate dioxygenase small subunit	*Cupriavidus basilensis* OR16	93/166	ZP_09623641.1
*benC* (002061)	BenC	339	Benzoate dioxygenase reductase subunit	*Cupriavidus metallidurans* CH34	77/339	YP_587015.1
*benD* (002063)	BenD	108	2-Hydro-1,2-dihydroxybenzoate dehydrogenase	*Ralstonia eutropha* JMP134	91/108	YP_298602.1
*pobA* (007971)	PobA2	389	4-Hydroxybenzoate 3-Monooxygenase	*Cupriavidus basilensis* OR16	92/389	ZP_09625369.1
*pobB* (001362)	PobA1	394	4-Hydroxybenzoate 3-Monooxygenase	*Pseudomonas putida* W619	92/394	YP_001748818.1
*pcaB* (001344)	PcaB	453	3-Carboxy-cis,cis-muconate cycloisomerase	*Streptomyces hygroscopicus* ATCC 53653	70/453	ZP_07293737.1
*pcaC1* (003089)	PcaC1	125	4-Carboxymuconolactone decarboxylase	*Pseudomonas fulva* 12-X	82/125	YP_004473183.1
*pcaC2* (005251)	PcaC2	139	4-Carboxymuconolactone decarboxylase	*Cupriavidus basilensis* OR16	90/139	ZP_09624697.1
*pcaD1* (000893)	PcaD1	280	3-Oxoadipate enol-lactonase	*Cupriavidus basilensis* OR16	86/280	ZP_09627578.1
*pcaD2* (006858)	PcaD2	273	3-Oxoadipate enol-lactonase	*Acidovorax avenae subsp. avenae* ATCC 19860	68/273	YP_004236298.1
*pcaF* (003109)	PcaF	400	*β*-Ketoadipyl CoA thiolase	*Cupriavidus basilensis* OR16	98/400	ZP_09627621.1
*pcaG* (002623)	PcaG	192	Protocatechuate 3,4-dioxygenase, *α* subunit	*Cupriavidus basilensis* OR16	88/192	ZP_09628855.1
*pcaH* (002624)	PcaH	241	Protocatechuate 3,4-dioxygenase, *β* subunit	*Cupriavidus basilensis* OR16	93/241	ZP_09628856.1
*pcaI1* (003111)	PcaI1	227	*β*-Ketoadipate succinyl-CoA transferase *α* subunit	*Ralstonia eutropha* H16	93/227	YP_728364.1
*pcaI2* (000701)	PcaI2	252	*β*-Ketoadipate succinyl-CoA transferase *α* subunit	*Burkholderia xenovorans* LB400	75/252	YP_554660.1
*pcaI3* (003203)	PcaI3	251	*β*-Ketoadipate succinyl-CoA transferase *α* subunit	*Bordetella petrii* DSM 12804	57/251	YP_001632201.1
*pcaI4* (002146)	PcaI4	233	*β*-Ketoadipate succinyl-CoA transferase *α* subunit	*Cupriavidus basilensis* OR16	95/233	ZP_09628963.1
*pcaJ1* (003112)	PcaJ1	215	*β*-Ketoadipate succinyl-CoA transferase *β* subunit	*Cupriavidus basilensis* OR16	95/215	ZP_09627620.1
*pcaJ2* (000700)	PcaJ2	220	*β*-Ketoadipate succinyl-CoA transferase *β* subunit	*Bordetella petrii* DSM 12804	68/220	YP_001633147.1
*pcaJ3* (003202)	PcaJ3	211	*β*-Ketoadipate succinyl-CoA transferase *β* subunit	*Cupriavidus basilensis* OR16	91/211	ZP_09626665.1
*pcaJ4* (002145)	PcaJ4	205	*β*-Ketoadipate succinyl-CoA transferase *β* subunit	*Cupriavidus basilensis* OR16	99/205	ZP_09628964.1
*pcaK* (003417)	PcaK	446	4-Hydroxybenzoate transporter	*Cupriavidus basilensis* OR16	93/446	ZP_09623076.1
*pcaL* (006326)	PcaL	399	3-Oxoadipate enol-lactone hydrolase/4-carboxymuconolactone decarboxylase	*Ralstonia eutropha* H16	74/399	YP_841800.1
*pcaQ* (002625)	PcaQ	266	Transcriptional regulator (LysR family)	*Cupriavidus basilensis* OR16	77/266	ZP_09628857.1
*pcaT* (001951)	PcaT	436	*β*-Ketoadipate transporter	*Pseudomonas syringae* pv. phaseolicola 1448A	78/436	YP_276146.1
*hcaA* (005714)	HcaA	280	*ρ*-Hydroxycinnamoyl-CoA hydratase/lyase	*Burkholderia glumae* BGR1	93/280	YP_002908324.1
*hcaB* (005963)	HcaB	397	Vanillin dehydrogenase	*Burkholderia thailandensis* MSMB43	86/397	ZP_02469116.1
*hcaC* (001154)	HcaC	620	Feruloyl-CoA synthase	*Azotobacter vinelandii* DJ	56/620	YP_002801316.1
*hcaD1* (003030)	HcaD1	395	Acyl-CoA dehydrogenase	*Cupriavidus basilensis* OR16	95/395	ZP_09626604.1
*hcaD2* (003645)	HcaD2	383	Acyl-CoA dehydrogenase	*Cupriavidus basilensis* OR16	95/383	ZP_09624899.1
*vanA1* (007066)	VanA1	351	Vanillate-O-demethylase oxygenase subunit	*Ralstonia solanacearum MolK2*	80/351	CAQ18072.1
*vanA2* (001443)	VanA2	340	Vanillate-O-demethylase oxygenase subunit	*Cupriavidus metallidurans* CH34	38/340	YP_587674.1
*vanB1* (007067)	VanB1	315	Vanillate-O-demethylase reductase subunit	*Pseudomonas syringae* pv. tomato str. DC3000	56/315	NP_792703.1
*vanB2* (001445)	VanB2	315	Vanillate-O-demethylase reductase subunit	*Halomonas elongata* DSM 2581	55/315	YP_003899010.1
*vanR* (7065)	VanR	258	Transcriptional regulator (GntR family)	*Cupriavidus basilensis* OR16	92/258	ZP_09625218.1
*hcaK* (002970)	HcaK	371	Hydroxycinnamate transporter	*Ralstonia eutropha* JMP134	84/371	YP_299063.1
*hcaR* (002968)	HcaR	164	Transcriptional activator (MarR family)	*Ralstonia eutropha* JMP134	75/164	YP_299066.1
*hcaX* (002969)	HcaX	404	Porin transmembrane protein	*Ralstonia eutropha* JMP134	88/404	YP_299064.1

a. Indicates the open reading frame number in the Additional file 1.

b. aa, number of amino acids.

The *pca* genes that are responsible for the protocatechuate branch of the *β-*ketoadipate pathway were dispersed throughout the genome of *C. basilensis* B-8 (Table 2), and this organization has not been previously reported for other bacteria. The *pcaIJF* genes encoding the enzymes required for the last two steps of the *β-*ketoadipate pathway clustered together. In some bacteria, the expression of the *pcaIJF* genes is induced by *β-*ketoadipate, which then activates IcIR family regulatory proteins PcaR/PcaQ. However, *pcaR* was found in another gene cluster and *pcaQ* was located in the vicinity of *pcaH* in *C. basilensis* B-8. It is not uncommon that transcriptional regulators control the expression of distal genes [29], though the exact mechanism of regulation for these genes requires further study. It was surprising that four copies of the *pcaI* and *pcaJ* genes were found in *C. basilensis* B-8 (Table 2), and the reason for this is still unknown. One striking aspect of the *pca* genes in *C. basilensis* B-8 is the presence of two copies of the *pcaC* and *pcaD* genes (which encode g-carboxymuconolactone decarboxylase and *β*-ketoadipate enol-lactone hydrolase, respectively) as well as a unique fused gene (*pcaL*) consisting of the *pcaC* and *pcaD* ORFs (Table 2). Sequence analysis of the *pcaL* gene revealed that the predicted C-terminal third was homologous to decarboxylases, whereas the N-terminal two thirds were homologous to enol-lactone hydrolases. Furthermore, DNA sequence data has revealed a remarkable feature of *catD* and *pacD* that encode the isozyme involved in the third from last step of the *C. basilensis* B-8 *β*-ketoadipate pathway (Figure 5). These two genes share only a 33% aa identity. Another striking aspect of the protocatechuate branch genes in *C. basilensis* B-8 is the presence of two 4-hydroxybenzoate 3-monooxygenase encoding genes, *pobA* and *pobB*. These two genes share 63.4% aa identity, indicating a distant evolutionary origin.

Ferulate and coumarate form a vast array of ether and ester bonds in lignin and suberin. In some bacteria, ferulate degradation follows a CoA-dependent non-*β*–oxidative pathway catalyzed by the feruloyl-CoA synthetase (HcaC) and enoyl-CoA hydratase/aldolase (HcaA) proteins, producing vanillin. Vanillin is further converted to protocatechuate via an aldehyde dehydrogenase (HcaB) and a demethylase (VanAB) [29]. The pathway for coumarate degradation into protocatechuate is similar to that of ferulate, which is conducted by PobA and/or PobB at the last step (Figure 5). Genes homologous to *hcaABCDKXR* have been identified in *C. basilensis* B-8. The *hcaABCD* genes are dispersed throughout the genome of *C. basilensis* B-8, whereas the *hcaKXR* genes are clustered together (Table 2). *hcaD* encoding an acyl-CoA dehydrogenase is not involved in the above biochemical pathway, but the protein could be responsible for a CoA-dependent *β*-oxidative pathway of ferulate degradation with HcaA and Aat (*β*-ketothiolase), as has been described in other organisms [28]. *hcaR*, *hcaK*, and *hcaX* encode a putative regulatory protein of the MarR family, a 3-hydroxyphenylpropionic acid transporter, and a putative porin of unknown function, respectively. Two *van* gene clusters were also identified in the genome of *C. basilensis* B-8 (Table 2), and one of them contained a transcriptional regulator of the GntR family (vanR). The *hca* gene cluster was not linked to the *van* cluster. A similar situation is also found in *P. putida* KT2440 and *Acinetobacter* sp. ADP1, and it has been suggested that this gene organization would facilitate the appearance of spontaneous van-deficient strains in natural *Acinetobacter* populations, which might allow the production of vanillate from ferulate as a chemical signal between plants and bacteria [35].

Cinnamate degradation via benzoate has been described in *Cupriavidus necator* JMP134 [29]. However, the enzymes that are responsible for the initial steps have not been characterized. Many similar intermediates including benzoate, produced during the process of cinnamate degradation by *C. basilensis* B-8 were observed on basis of our GC-MS analysis (date not shown). Accordingly, we could predict that cinnamate was also degraded through benzoate (Figure 5). The study of the enzymes involved in the first five steps of this pathway is currently in progress.

### Phenol degradation pathways

Bacterial catabolic pathways for phenol and its derivatives have been studied extensively in *C*. *necator* JMP134 [29]. Genomic analysis of *C. basilensis* B-8 showed that all orthologous genes were present except *phlX*, which encodes a relatively hydrophobic protein. Phenol and its derivatives metabolized via the methylcatechol *ortho* ring-cleavage pathway (enzymes encoded by *mml* genes) and the catechol *meta* ring-cleavage pathway (enzymes encoded by *phl* genes) in *C. basilensis* B-8 are shown in Figure 5, and the involved enzymes are listed in Table 3. The *mml* genes organized in a single cluster. The *mmlJIHGFRL* is maintained in the *mml* clusters of *C. basilensis* B-8 (Table 3) and *C*. *necator* JMP134. Similar to *C*. *necator* JMP134, no putative gene encoding an isoenzyme of *β*-ketoadipate enollactone hydrolase was found in the *mml* gene cluster of *C. basilensis* B-8. This point supports the idea that 4-methyl-*β*-ketoadipate enollactone is not further metabolized through a classical *β*-ketoadipate pathway. The next step in the reaction process still requires further study. 

**Table 3:** Genes encoding catabolic pathways for phenols and its derivatives degradation

**Gene (orf no.)**^**1**^	**Gene product**	**Size (aa)**^**2**^	**Function**	**Organism**	**% Identity/aa**	**Accession no.**
*phlB* (007044)	PhlB	310	Catechol-2,3-dioxygenase	*Ralstonia* sp. KN1	97/310	BAA84125.1
*phlC* (007045)	PhlC	484	2-Hydroxymuconic semialdehyde	*Cupriavidus basilensis* OR16	96/484	ZP_09625234.1
*phlD1* (001164)	PhlD1	279	2-Hydroxymuconic semialdehyde hydrolase	*Azotobacter vinelandii* DJ	72/279	YP_002801325.1
*phlD2* (002956)	PhlD2	291	2-Hydroxymuconic semialdehyde hydrolase	*Burkholderia thailandensis* MSMB43	69/291	ZP_02463428.1
*phlE* (007046)	PhlE	260	2-Hydroxypent-2,4-dienoate hydratase	*Ralstonia eutropha* H16	91/260	YP_728710.1
*phlF* (008422)	PhlF	313	Acetaldehyde dehydrogenase (acylating)	*Pseudomonas resinovorans*	82/313	NP_758577.1
*phlG* (008421)	PhlG	335	4-Hydroxy-2-oxovalerate aldolase	*Dechloromonas aromatica* RCB	82/335	YP_286438.1
*phlH* (007047)	PhlH	262	4-Oxalocrotonate decarboxylase	*Cupriavidus basilensis* OR16	95/262	ZP_09625232.1
*phlI* (007048)	PhlI	63	4-Oxalocrotonate isomerase	*Cupriavidus basilensis* OR16	90/63	ZP_09625231.1
*phlK* (007037)	PhlK	72	Phenol hydroxylase subunit	*Ralstonia* sp. KN1	93/72	BAA84118.1
*phlL* (007038)	PhlL	331	Phenol hydroxylase subunit	*Ralstonia* sp. KN1	95/331	BAA84119.1
*phlM* (007039)	PhlM	94	Phenol hydroxylase subunit	*Cupriavidus basilensis* OR16	95/94	ZP_09625240.1
*phlN* (007040)	PhlN	504	Phenol hydroxylase subunit	*Ralstonia* sp. KN1	97/504	BAA84121.1
*phlO* (007041)	PhlO	119	Phenol hydroxylase subunit	*Ralstonia* sp. KN1	90/119	BAA84122.1
*phlP* (007042)	PhlP	355	Phenol hydroxylase subunit	*Cupriavidus basilensis* OR16	93/355	ZP_09625237.1
*phlQ* (007043)	PhlQ	111	Ferredoxin	*Ralstonia* sp. KN1	89/111	BAA84124.1
*phlR* (007035)	PhlR	571	Phenol hydroxylase regulator protein	*Cupriavidus basilensis* OR16	95/571	ZP_09625495.1
*mmlF* (000701)	MmlF	252	Oxoadipate-CoA Transferase *α* subunit	*Ralstonia eutropha* H16	88/252	NP_943023.1
*mmlG* (000700)	MmlG	220	Oxoadipate-CoA Transferase *β* subunit	*Cupriavidus necator* N-1	91/220	YP_004687736.1
*mmlH* (000699)	MmlH	428	Muconolactone transporter	*Ralstonia eutropha* JMP13	86/428	YP_295715.1
*mmlI* (000698)	MmlI	112	4-Methylmuconolactone methylisomerase	*Ralstonia eutropha* H16	83/112	NP_943020.1
*mmlJ* (000697)	MmlJ	87	Methylmuconolactone isomerase	*Cupriavidus necator* N-1	83/87	YP_004687733.1
*mmlL* (000704)	MmlL	296	Hypothetical protein (Zn-dependent hydrolases)	*Ralstonia eutropha* H16	16/296	NP_943025.1
*mmlR* (000703)	MmlR	304	Transcriptional activator (LysR family)	*Ralstonia eutropha* H16	91/304	NP_943024.1

a. Indicates the open reading frame number in the Additional file 1.

b. aa, number of amino acids.

Although the *mml* genes are clustered together, the *phl* genes were organized in two different clusters. *phlGF* encoding the 4-hydroxy-2-ketovalerate aldolase and the aldehyde dehydrogenase that catalyzes the final steps of the meta ring-cleavage pathway (Figure 5) are separated from the rest of the other meta ring-cleavage pathway genes. A similar arrangement is also present in *C*. *necator* JMP134, though one difference is that two copies of *phlD* (which encodes a 2-hydroxymuconic semialdehyde hydrolase) were not found in either of these two gene clusters (Table 3).

### The gentisate pathway for catabolism of salicylate and 3-hydroxybenzoate

Salicylate is generated from benzoic acid hydroxylation or trans-cinnamic acid side chain *β*-oxidation in plants. The catabolism of salicylate into catechol by salicylate 1-hydroxylase, a flavoprotein monooxygenase, or by a three-component protein has been described in several bacteria [29]. Genetic analysis of the *C. basilensis* B-8 genome showed seven genes (data not shown) with high identity to those from *Pseudomonas* and *Acinetobacter* strains. An alternative route of salicylate degradation, via a gentisate intermediate, is initiated by a multicomponent oxygenase (salicylate-5-hydroxylase), as has been reported in *Ralstonia* sp. U2 [36]. The putative LysR-type transcriptional regulator encoding the *hybR* gene, large and small subunits of the oxygenase encoding *hybB* and *hybC* genes, as well as the ferredoxin encoding *hybD* gene were organized in a single gene cluster (Table 4), which shows a significant similarity with that from *C. necator* JMP134. Further study is required to develope our understanding of how salicylate is converted into catechol in *C. basilensis* B-8.

**Table 4:** Genes encoding gentisate pathways and peripheral reactions

**Gene (orf no.)**^**1**^	**Gene product**	**Size (aa)**^**2**^	**Function**	**Organism**	**% Identity/aa**	**Accession no.**
*hybR* (000498)	HybR	306	LysR-like regulator protein	Ralstonia solanacearum UW551	80/306	ZP_00946271.1
*hybA* (000499)	HybA	328	Putative ferredoxin oxidoreductase	*Ralstonia solanacearum* Po82	70/328	YP_006030105.1
*hybB* (000500)	HybB	418	Salicylate-5-hydroxylase large oxygenase component	*Ralstonia solanacearum* UW551	83/418	ZP_00946273.1
*hybC* (000501)	HybC	157	Salicylate-5-hydroxylase small oxygenase component	*Ralstonia solanacearum* MolK2	72/157	CAQ37385.1
*hybD* (000502)	HybD	103	Salicylate 5-hydroxylase ferredoxin component	*Achromobacter xylosoxidans* A8	66/103	YP_195869.1
*mhbR* (003412)	MhbR	319	Transcriptional regulator (LysR family)	*Cupriavidus necator* N-1	77/316	YP_004681033.1
*mhbD* (003413)	MhbD	348	Gentisate 1,2-dioxygenase	*Cupriavidus basilensis* OR16	95/348	ZP_09623073.1
*mhbH* (003414)	MhbH	232	Fumarylacetoacetate hydrolase	*Cupriavidus basilensis* OR16	91/232	ZP_09623074.1
*mhbI* (003415)	MhbI	214	Maleylpyruvate isomerase	*Ralstonia eutropha* H16	76/214	YP_729032.1
*mhbM* (003416)	MhbM	413	3-Hydroxybenzoate-6-hydroxylase	*Burkholderia multivorans* CGD1	82/413	ZP_03585707.1
*mhbT* (003417)	MhbT	446	Putative 3-hydroxybenzoate transporter	*Burkholderia* sp. NCIMB 10467	59/446	ABW22835.1

a. Indicates the open reading frame number in the Additional file 1.

b. aa, number of amino acids.

3-hydroxybenzoate is degraded through gentisate by 3-hydroxybenzoate-6-hydroxylase (3H6H) or through protocatechuate by 3-hydroxybenzoate-4-hydroxylase (3H4H) in *Comamonas testosteroni*[37] and *Bacillus* sp. [38]. No homologue of the 3H4H gene was found in the genome of *C. basilensis* B-8. However, a gene (*mhbM*) was identified with 82.0% aa identity with that of the 3H6H gene sequence from *Burkholderia multivorans* CGD1 (Table 4).

The gentisate pathway (Figure 5) is initiated by a gentisate-1, 2-dioxygenase (MhbD), which cleaves the aromatic ring to form maleylpyruvate. Maleylpyruvate could be further degraded by maleylpyruvate hydrolase or by maleylpyruvate isomerase, central metabolites of the Krebs cycle. Genomic analysis indicated the presence of an *mhb* gene cluster in the genome of *C. basilensis* B-8. The *mhbR* and *mhbT* genes were located upstream of the *mhb* gene cluster and encoded a LysR family regulator protein and 3-hydroxybenzoate transporter, respectively (Table 4). *mhbI* and *mhbH* are homologous to genes that encode maleylpyruvate isomerase and fumarylpyruvate hydrolase in *C. necator* JMP134 [29], respectively. Together, these observations suggested that gentisate is metabolized by a glutathione-dependent pathway in *C. basilensis* B-8, as it is in *C. necator* JMP134.

## Conclusions

This study demonstrated that *C. basilensis* B-8 could be a useful tool for lignin utilization. The great capability for KL degradation by this strain was confirmed. The maximum degradation rate was 44.4% at the initial concentration of 2 g L^-1^ after 7 days of incubation. High activity of MnP and Lac as well as the presence of many intermediates was observed during the degradation progress. Comprehensive and systematic whole genomic analysis of bacterial lignin degradation pathways was also performed via sequencing and analysis of the *C. basilensis* B-8 genome, and many genes related to lignin degradation were identified. Three major pathways for lignin degradation were reconstructed via genomic analysis.

## Methods

### Bacterial strain and cultural conditions

A large number of bamboo slips of Kingdom Wu during the Three-Kingdoms Dynasty, were unearthed from Zoumalou, Hunan province, China, and stored in the Bamboo Slips Museum of Changsha, Hunan Province. The bamboo slips are rare cultural relic of Chinese history. After being unearthed, the bamboo slips were severely eroded by microorganisms. The surface layer of the eroded bamboo slips consisted of a translucent film material, and this was brittle and easily removed. It was likely that lignin and cellulose in the bamboo slips could be degraded by microorganisms present in the samples. The bamboo slips were kept in water to prevent air oxidation, and the bacterial strain *C. basilensis* B-8 was obtained from the steeping fluid of the eroding bamboo slips. Cells were grown in the Luria-Bertani broth medium and incubated at 30°C with shaking at 120 rpm to an OD_600_ (optical density at 600 nm) of the inoculums reached approximately 1.0. Then, 2 ml of culture was aseptically inoculated into three parallel culture flasks containing 100 ml sterile KL mineral salt medium (3 g KL, 2 g [NH_4_]_2_SO_4_, 1 g K_2_HPO_4_, 1 g KH_2_PO_4_, 0.2 g MgSO_4_, 0.1 g CaCl_2_, 0.05 g FeSO_4_, and 0.02 g MnSO_4_ in 1 L distilled water, pH 7.0). The flasks were incubated at 30°C with shaking at 120 rpm for 7 days. The temperature, pH, and the contents of KL were properly adjusted according to the experimental requirements. Uninoculated medium was used as a control. The KL (molecular weight of approximately 10 000) used in these experiments was purchased from Sigma Aldrich (St. Louis, MO, USA).

### Assessment of aromatic compounds as sole carbon and energy sources for *C*. *basilensis* B-8

The aromatic compounds including 4-hydroxycinnamate (coumarate) ; ferulate;  cinnamate;  benzoate; 3, 4- dihydroxybenzoate (protocatechuate); vanillate; phenol; 4-methylcatechol; 2-methylphenol; salicylate;  3-hydroxybenzoate;  ethyl  salicylate;  and 3-hydroxbenzylalcohol were added into each flask, which contain 50 ml of mineral salt medium described above, at a concentration of 100 mg L^-1^. The strain that was grown for about 12 h in 200 mL of LB medium was harvested by centrifugation at 10 000 rpm for 10 min, washed three times with sterilized deionized water, and resuspended in 50 ml of sterilized deionized water. Samples (1 ml) were added into each flask with shaking at 150 rpm. A flask without any aromatic compounds was used as a control.

### Bacterial growth and COD measurements

The rate of *C*. *basilensis* B-8 growth was determined by measuring the OD_600_ of cultured samples using a spectrophotometer (U-4100; Hitachi, Tokyo, Japan). The control and cultured samples (liquid samples) were centrifuged at 12 000 g for 10 min to remove biomass. The appropriate volume of the supernatant was introduced into digestion solution (100–1000 mg · L^-1^) containing potassium dichromate, sulfuric acid, and mercuric sulfate. The mixture was then incubated for 15 min at 165°C ± 2°C in a COD reactor (Model 45600; Hach Company, Loveland, CO, USA). The COD concentration was measured colorimetrically using a spectrophotometer.

### Enzyme assays

A total of 1 ml of the control or cultured samples were centrifuged at 12 000 rpm for 5 min to remove suspended solids. Cell-free supernatant was used as the enzyme source to determine the activity of laccase, lignin peroxidase, and manganese peroxidase. Laccase activity was determined by monitoring the oxidation of ABTS at 420 nm (ε420 = 36000 mol^-1^ cm^-1^) [39]. Lignin peroxidase activity was determined by monitoring the peroxide-dependent oxidation of 2 mM veratryl alcohol to veratraldehyde at 310 nm (ε310 = 9 300 mol^-1^ cm^-1^) [40]. Manganese peroxidase activity was determined by monitoring the oxidation of 2, 6-DMP to coerulignone at 469 nm (ε469 = 49600 mol^-1^ cm^-1^) [41].

### GC-MS analysis

The control and cultured samples containing 3 g L^-1^ KL were periodically withdrawn and centrifuged at 12 000 rpm for 10 min. Supernatants were acidified to pH 2.0 with 6 mmoL L^-1^ HCL and then extracted with an equal volume of ethyl acetate. Three portion of extracts were collected, dewatered over anhydrous Na_2_SO_4_, filtered though filter paper, and evaporated at 40°C under vacuum on a rotary vacuum evaporator. Then, 0.1 ml dioxane and 0.01 ml pyridine were added in the samples followed by silylation with 0.05 ml TMS. The mixture was heated at 60°C for 15 min with periodic shaking to dissolve the residues. GC-MS analysis of organic extracts was conducted using the method reported previously [18]. The identification of low molecular weight compounds as TMS derivatives derived from bacterial degradation was performed by comparing their mass spectra with that of the National Institute of Standards and Technology (NIST) library available in the instrument and also by comparing the retention time (RT) with those of available authentic compounds.

### Sequencing and analysis of the *C*. *basilensis* B-8 genome

The genomic DNA of *C*. *basilensis* B-8 was extracted and purified using a TIANamp Bacteria DNA Kit (Tiangen Biotech, Beijing, China) and then used for sequencing. A total of 495 Mb of clean data from an insert size library (500 bp) was generated for the strain using a high-throughput HiSeq2000 paired-end strategy (Illumina, San Diego, CA, USA). Contigs were assembled and aligned using SOAPdenovo 1.05 software developed by the Beijing Genomics Institute. A total of 8448 coding sequences (CDSs) were predicted using Glimmer3.0, rRNAmmer, tRNAscan, and Rfam software [42-45]. Before the manual annotation of the predicted genes (Additional file 1), automatic annotation was computed using different tool results as follows: similarity searches were performed against different databases including KEGG, Swiss-Prot, TrEMBL, and NR using BLASTall v.2.2.21 software. The annotation results are available in Additional file 2. The aa sequences of the ORFs were compared with those present in finished and unfinished microbial genome databases using the TBLASTN algorithm at the NCBI server (http://www.ncbi.nlm.nih.gov/blast/blast.cgi). Nucleotide and protein sequence similarity searches were also performed using BLAST programs at the BLAST server of NCBI. Pairwise and multiple protein sequence alignments were made with the ALIGN and CLUSTALW programs, respectively, available at the INFOBIOGEN server (http://www.infobiogen.fr/services/menuserv.html). A number of genes responsible for degradation of lignin and its derivatives were identified. The aa sequence of the products from the genes responsible for lignin-related aromatic compound degradation from other reported bacterial strains were compared with the translated genome of *C. basilensis* B-8. By this approach, we were able to identify the predicted genes responsible for the lignin degradation pathways. The Whole Genome Shotgun project has been deposited at DDBJ/EMBL/GenBank under the accession AKXR00000000. The version described in this paper is the first version, AKXR01000000.

## Abbreviations

KL: Kraft lignin; MnP: Manganese peroxidase; Lac: Laccase; LiP: Lignin peroxidase; OD: Optical density; COD: Chemical oxygen demand; TIC: Total ion chromatograph; ORFs: Open reading frames; aa: Amino acid; CDSs: Coding sequences; 3H6H: 3-hydroxybenzoate-6-hydroxylase; 3H4H: 3-hydroxybenzoate-4-hydroxylase; OD_600_: Optical density at 600 nm.

## Competing interests

The authors declare that they have no competing interests.

## Authors’ contributions

YS designed the study, performed the experiments, analyzed the results, and wrote the manuscript. LYC co-designed the study. TCJ and YZH revised the manuscript. HZ and YZ carried out the GC-MS experiments. RHC and YHC contributed to writing of the manuscript. All authors read and approved the final manuscript.

## Supplementary Material

Additional file 1 **Open reading frames in *Cupriavidus basilensis *B-8genome.** Click here for file

Additional file 2 ***Cupriavidus basilensis *B-8 genes annotation.** Click here for file
